# Inflammation and Immune Pathways in Myopia: An Overview on Pathomechanisms and Treatment Prospects

**DOI:** 10.1007/s12016-025-09094-7

**Published:** 2025-11-05

**Authors:** Jing Zhang, Koju Kamoi, Yuan Zong, Mingming Yang, Yaru Zou, Kyoko Ohno-Matsui

**Affiliations:** Department of Ophthalmology & Visual Science, Graduate School of Medical and Dental Sciences, Institute of Science Tokyo, 1-5-45 Yushima, Bunkyo-Ku, Tokyo, 113-8510 Japan

**Keywords:** Myopia, Inflammation, Immune microenvironment, Cytokines, Scleral remodeling, Anti-inflammatory therapy

## Abstract

Myopia represents a growing global public health challenge, characterized by increasing prevalence and associated complications such as myopic macular degeneration and retinal detachment. Although genetic and environmental factors are well-recognized contributors, emerging evidence supports a pathological link between inflammation and myopia progression. Epidemiological studies indicate a higher incidence of myopia among individuals with systemic or ocular inflammatory conditions. Inflammation perturbs the ocular immune microenvironment by upregulating pro-inflammatory cytokines and matrix metalloproteinase-2, thereby accelerating extracellular matrix (ECM) degradation and scleral remodeling, which culminates in axial elongation. Conversely, excessive axial elongation in high myopia triggers choroidal microvascular dysfunction, tissue hypoxia, and disruption of the blood-retinal barrier, leading to elevated inflammatory cytokines in the aqueous humor and vitreous, thereby raising the possibility of a self-perpetuating loop. Anti-inflammatory agents, including diacerein, resveratrol, and lactoferrin, have demonstrated therapeutic potential in experimental models by modulating inflammatory pathways, reducing pro-inflammatory cytokines, and preserving ECM integrity. However, their clinical efficacy and long-term safety require further validation. Elucidating the complex interplay between inflammation and myopia is pivotal for the development of targeted interventions, moving the focus of myopia management beyond optical correction towards disease-modifying strategies.

**Graphical Abstract:**

Targeting inflammation to disrupt the vicious cycle and unlock new myopia treatments (by Figdraw).

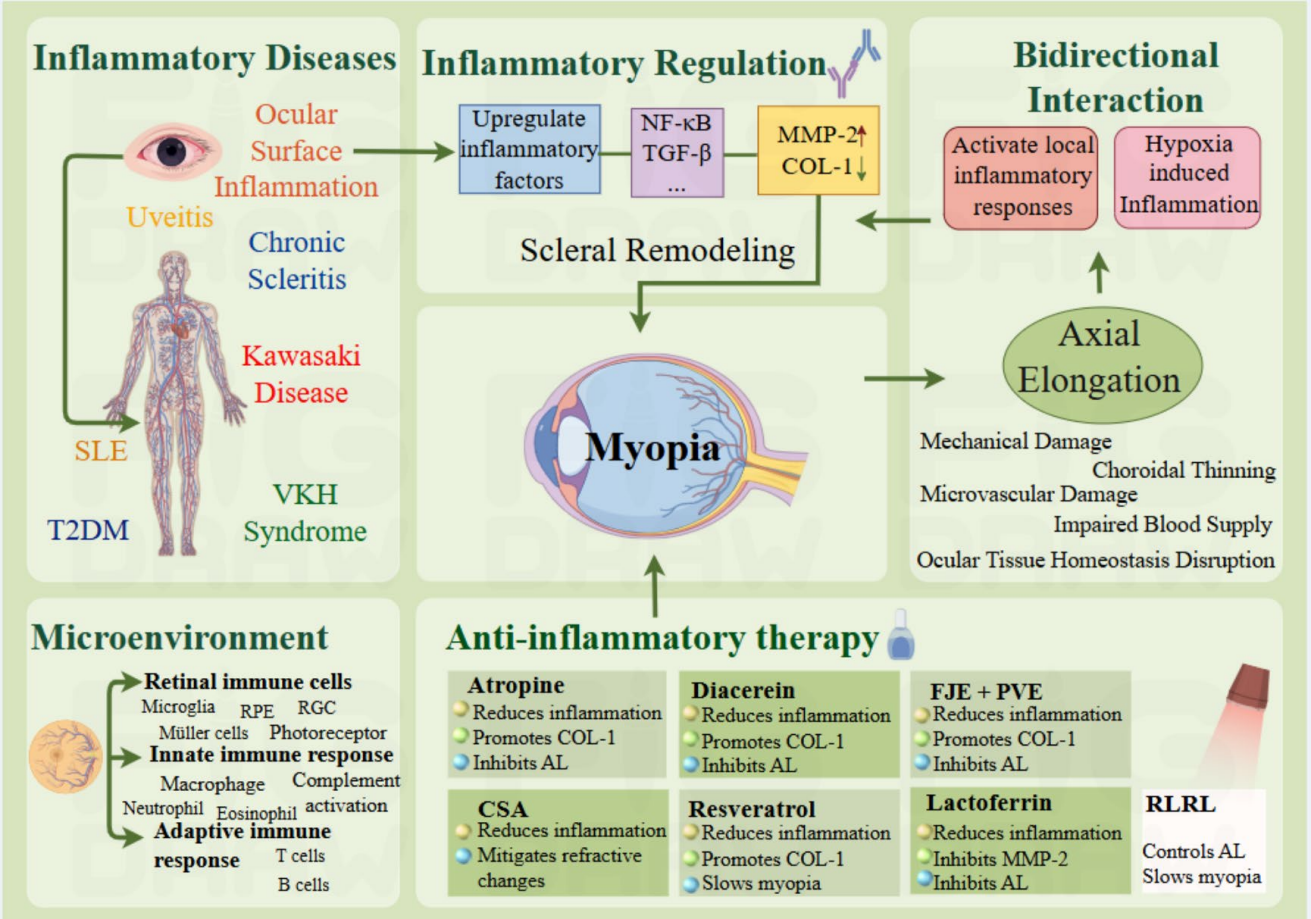

## Introduction

Myopia has emerged as a significant global public health challenge. Epidemiological studies estimate that its current global prevalence exceeds 28%, with nearly 50% of the population expected to be affected by 2050 [1]. In Asia, the prevalence is significantly higher, with approximately 80% of children and adolescents developing myopia by the end of their schooling [2]. Beyond impairing visual function, myopia markedly increases the risk of ocular diseases, including glaucoma and retinopathy [3, 4]. Given these staggering epidemiological trends and associated ocular comorbidities, developing effective prevention and treatment strategies represents an urgent priority in ophthalmic research and public health policy.

Conventional understanding attributes myopia pathogenesis to the complex interplay between genetic susceptibility [5] and environmental factors, particularly prolonged near-work activities and limited outdoor exposure [6, 7]. In addition to genetic predisposition and environmental influences, several complementary hypotheses have been proposed to explain the development of myopia. The optical defocus theory posits that peripheral hyperopic defocus induces compensatory axial elongation [8]. The dopamine hypothesis highlights that reduced retinal dopamine signaling, associated with limited exposure to outdoor light, may contribute to myopia development [9, 10]. Furthermore, behavioral and lifestyle factors such as prolonged near work, increased screen time, and insufficient outdoor activity have been consistently associated with the rising prevalence of myopia [11]. A more integrated understanding of these diverse contributors is essential for informing comprehensive prevention and management strategies.

Recently, increasing attention has been given to the potential role of inflammation and immune dysregulation in driving myopia progression through the effects on scleral remodeling and axial elongation. As an immune-privileged organ, the eye regulates immune responses through tolerance mechanisms, minimizing inflammatory damage to essential structures [12]. The onset of myopia is associated with changes in the ocular immune microenvironment, including immune cell activation and elevated inflammatory factors. These changes may accelerate myopia progression by influencing scleral remodeling. Studies show that inflammatory factors like tumor necrosis factor-alpha (TNF-α), interleukin-6 (IL-6), and nuclear factor kappa B (NF-κB) are significantly elevated in myopia models, suggesting a key role for the immune response in myopia development [13]. In addition to ocular inflammatory conditions, certain subtypes of uveitis such as juvenile idiopathic arthritis-associated uveitis, multifocal choroiditis, and punctate inner choroiditis have been reported to be associated with myopic progression [14]. Furthermore, elevated inflammatory cytokines from systemic inflammatory diseases may contribute to myopia progression through disruption of the blood-retinal barrier (BRB) [15]. In adolescents, incomplete ocular development, including dynamic changes in lens accommodation, ciliary muscle instability, and scleral fragility, increases susceptibility to intraocular environmental fluctuations, predisposing them to myopia [16]. Therefore, early detection and intervention in myopia progression are crucial for adolescents.

This review aims to provide a comprehensive overview of the ocular immune microenvironment, explore the influence of inflammatory diseases, and elucidate the mechanisms linking inflammation and myopia. Specifically, we emphasize the translational relevance of immunological insights by discussing potential anti-inflammatory therapeutic strategies, including both conventional and emerging agents. We also propose an integrative mechanistic framework that connects immune activation with scleral remodeling through barrier disruption, cytokine cascades, and immune cell recruitment. In addition, we examine how developmental characteristics of the adolescent eye, such as accommodation instability and scleral biomechanical plasticity, may contribute to increased susceptibility to inflammatory dysregulation. By incorporating these perspectives and synthesizing recent findings, this review aims to provide a conceptual and practical foundation for the clinical application of immune-modulating strategies in myopia management.

## The Immune Microenvironment in Myopia

Myopia development is not merely a refractive alteration but involves complex regulation of the immune microenvironment. Maintaining immune homeostasis within the eye is critical for normal visual function; disruption of this balance can lead to axial elongation and myopia progression [17]. Therefore, understanding the correlation between inflammation and myopia in the intraocular immune microenvironment is essential. These regulatory processes influence the development of myopia by modulating choroidal and scleral remodeling [18].

### Retinal Resident Immune Cells

The retina contains a variety of resident immune cells, which are essential in maintaining homeostasis, detecting pathogens, and regulating inflammatory responses. Among these cells, glial, retinal ganglion cells (RGCs), photoreceptor cells, and retinal pigment epithelial (RPE) cells contribute significantly to immune regulation. Glial cells, including microglia, astrocytes, and Müller cells, are primarily located in the inner retina [19]. The microglial activation leads to the release of inflammatory mediators that impact the function of the RPE [18], and single-cell RNA sequencing in mice revealed increased microglial activity in high myopic retina [20]. Astrocytes and Müller cells support retinal structure [21]. Astrocyte proliferation in myopia is linked to retinal vascular changes and neuronal signaling, with studies showing reduced blood supply and capillary loss in myopic retinas [22, 23]. PRSS56, produced by Müller cells, modulates the structural organization of their endfeet at the inner limiting membrane [24], which, along with the transmembrane glycoprotein MFRP, may contribute to axial elongation [25].

**Fig. 1:**
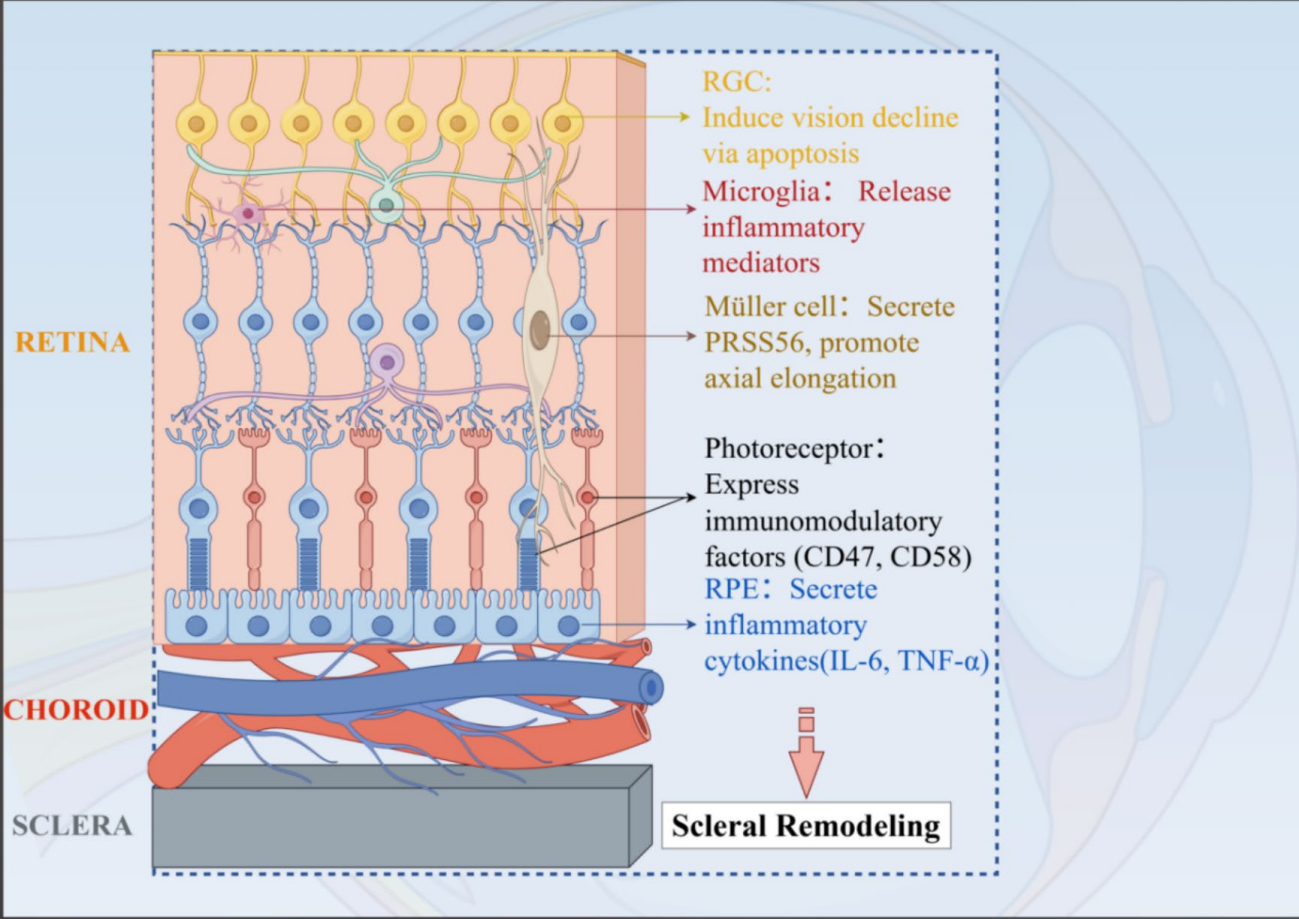
Overview of retinal cells in immune regulation and myopia progression. Schematic overview of key retinal cell types involved in immune regulation and their roles in myopia. Retinal ganglion cells (RGCs) undergo apoptosis in response to chronic inflammation, contributing to visual decline. Microglia, the resident immune cells of the retina, secrete pro-inflammatory mediators that disrupt retinal homeostasis. Müller glia span the full thickness of the retina and secrete serine protease PRSS56, which has been implicated in axial elongation. Photoreceptors express immunomodulatory molecules such as CD47 and CD58, shaping immune responses within the subretinal space. Retinal pigment epithelial (RPE) cells maintain ocular immune privilege and secrete cytokines including IL-6, contributing to local inflammation. Inflammatory signaling further promotes scleral remodeling, establishing a mechanistic link between immune dysregulation and progressive myopia. Illustration created with Figdraw. Abbreviations: CD47, cluster of differentiation 47; CD58, cluster of differentiation 58; IL-6, interleukin-6; PRSS56, serine protease 56; RGC, retinal ganglion cell; RPE, retinal pigment epithelium; TNF-α, tumor necrosis factor-alpha.

Overextension of the eyeball in high myopia leads to mechanical stretching and deformation of the retina, particularly affecting the RGCs located in the nerve fiber layer. Furthermore, chronic inflammation contributes to RGC apoptosis, leading to a progressive decline in visual function [26]. Photoreceptor cells express a variety of immunomodulatory factors, such as CD47 and CD58, which regulate the immune microenvironment in the subretinal space in cooperation with the RPE [27]. RPE cells are the key component of the outer blood-retinal barrier and constitute an ocular “immune privilege” by deviating from or suppressing destructive inflammation [28]. Furthermore, RPE cells contribute to myopia progression by secreting inflammatory cytokines such as TNF-α and IL-6, which are upregulated in allergic conjunctivitis and have been shown to disrupt epithelial barriers and promote ocular tissue remodeling leading to axial elongation [29]. The diverse roles of these retinal cell types in immune regulation and their involvement in myopia pathogenesis are summarized in Fig. 1.

### Intraocular Inflammatory Immunomodulation

**Fig. 2:**
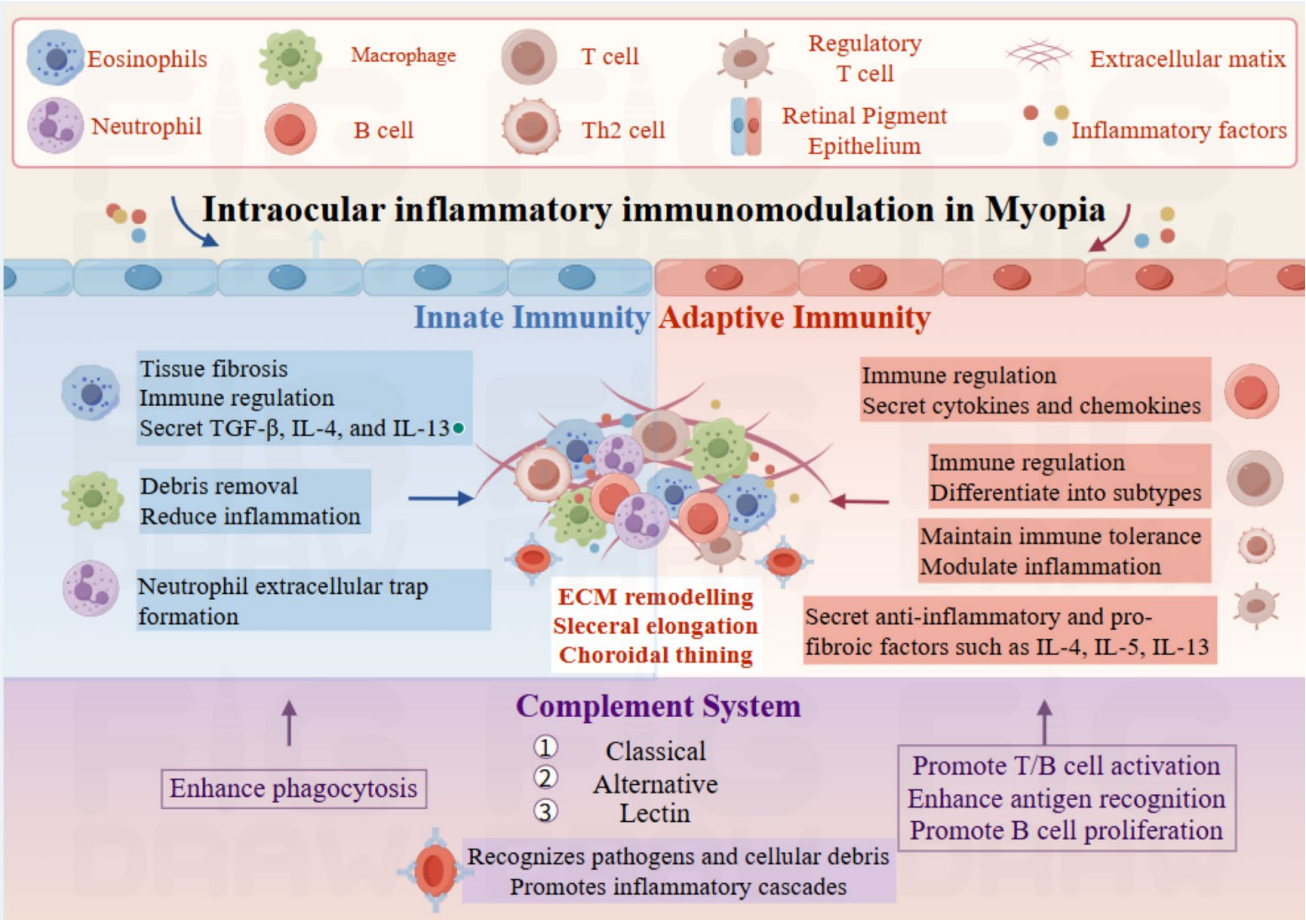
Intraocular inflammatory immunomodulation in myopia. Toll-like receptors (TLRs) expressed on retinal pigment epithelial (RPE) cells initiate innate immune responses upon recognition of pathogen- or damage-associated molecular patterns. Innate immune cells, including neutrophils, macrophages, and eosinophils, contribute to tissue remodeling, debris clearance, and extracellular trap formation. Adaptive immunity involves T cells, B cells, and regulatory T cells, which regulate inflammation through cytokine and chemokine secretion, differentiation, and maintenance of immune tolerance. The complement system enhances phagocytosis and facilitates activation and antigen presentation to both T and B cells. These immune processes collectively contribute to extracellular matrix (ECM) remodeling, scleral elongation, and choroidal thinning in myopia. Illustration created with Figdraw. Abbreviations: B cell, B lymphocyte; ECM, extracellular matrix; IL, interleukin; NET, neutrophil extracellular trap; RPE, retinal pigment epithelium; T/B cell, T and B lymphocyte; TGF-β, transforming growth factor-beta; Th2 cell, type 2 helper T cell; TLR, toll-like receptor; VEGF, vascular endothelial growth factor

Despite its immune privilege, the eye undergoes complex, multilayered inflammatory immune regulation. As shown in Fig. 2, RPE cells, as key effectors of immune regulation, dynamically coordinate both innate and adaptive immune responses via the toll-like receptor signaling pathway, complement cascade, and antigen presentation [30].

Innate immune cells, including macrophages, neutrophils, and eosinophils, play crucial roles in the ocular immune response. The intraocular microenvironment regulates macrophage development, differentiation, and activity [31, 32]. RPE cells enhance macrophage debris removal by modulating their phenotype and function [33], while glial cell activation mitigates inflammation by promoting the M2 macrophage phenotype [34]. In a 2025 study, inhibition of macrophage extracellular traps releases suppressed lens-induced myopia progression in mice, highlighting their potential role in refractive development [35]. Neutrophil extracellular traps, released by activated neutrophils, have been identified in multiple ocular surface diseases such as dry eye disease (DED), infectious keratitis, and autoimmune uveitis, where they mediate epithelial injury, promote fibrosis, and sustain inflammatory responses [36]. Additionally, eosinophils modulate ocular inflammation through transforming growth factor-beta (TGF-β)1-mediated fibroblast activation and chemotactic signaling in cooperation with IL-4 and IL-13 [37].

Adaptive immune cells are specifically activated under conditions of chronic inflammation in the retina. Analysis of infiltrating immune cells has shown a significant enrichment of T and B cells in myopic corneas [38], with sequential synthesis of pro-inflammatory mediators recruiting or activating T cells. Different T cell subpopulations secrete a variety of mediators and growth factors that regulate the intraocular immune microenvironment. For example, Type 2 helper T (Th2) cells secrete anti-inflammatory and pro-fibrotic factors such as IL-4, IL-5, and IL-13, which promote the overdeposition of key proteins, such as collagen and matrix metalloproteinases, during extracellular matrix (ECM) remodeling [39]. Regulatory T cells (Tregs) are essential for maintaining immune tolerance and modulating inflammatory responses [40]. A recent study indicates that the balance between T helper 17 (Th17) cells and Tregs plays a pivotal role in controlling intraocular inflammation, as evidenced by their opposing trends in autoimmune uveitis patients [41]. Furthermore, B lymphocytes secrete cytokines and chemokines that modulate pro-inflammatory immune responses [42].

The complement system bridges innate and adaptive immunity by rapidly recognizing pathogens, facilitating phagocytosis, and initiating inflammatory responses through the classical, alternative, and lectin pathways [43]. These pathways interact with other immune signaling mechanisms, forming a complex regulatory network [44]. In adaptive immunity, complement activation products regulate T cell activation and differentiation, enhance antigen recognition by B cells, and stimulate B cell proliferation [45, 46]. The expression of complement regulatory factors is crucial for maintaining immune homeostasis [47]. These complex and multilayered immunological interactions within the retina are visually summarized in Fig. 2, highlighting the coordinated roles of innate and adaptive immune mechanisms in myopic progression.

### Immune Biomarkers Associated with Myopia

With the deepening of immune microenvironment research, the role of inflammation-related factors as critical biomarkers in myopia has gradually gained attention. The correlation between inflammation-related factors and myopia is summarized in Table 1.

**Table 1:** Potential inflammation-related biomarkers in myopia

Biomarkers	Myopia type	Sample source	Detection	Results in patients/models
TGF-β2	High myopia	Aqueous humor	ELISA	Higher concentration Positively correlated with AL [48, 49]
GDF-15, HGF, PDGF-AA	High myopia	Aqueous humor	Bio-Plex ProTM multiplex bead-based immunoassay	Higher concentration [50]
IL-6	High myopia	Aqueous humor Vitreous humor	ELISA Human Magnetic Luminex Assay	Higher concentration Positively correlated with AL [51, 52]
IL-8	Myopia High myopia	Aqueous humor	Ella™ and ELISA	Higher concentration Positively correlated with AL [53]
IL-1β	High myopia	Aqueous humor	Human Magnetic Luminex Assay	Higher concentration [52]
MCP-1	High myopia	Aqueous humor	Suspension cytokine array Western blot assay ELISA	Higher concentration [54] Positively correlated with AL [49]
IFN-γ, eotaxin, IP-10, MIP-1α	High myopia	Vitreous humor	Bio-Plex Pro™ Human Cytokine 27-plex Assay Luminex Human Magnetic Assay	Higher concentration [55]
IL-1β, IL-6, TNF-α	Myopia	Aqueous humor	Cytometric bead array	No significant correlation with AL [57]
TGF-β, MMP-2, IL-6, IL-8, TNF-α, MCP-1	Experimental myopia model	FDM model MFD model	Immunohistochemistry Quantitative PCR Immunofluorescence staining Western Blot Analysis	Higher concentration [13, 56]
sICAM-1	Progressive myopia	Aqueous humor	ELISA	Positively correlated with AL [49]
PLG	High myopia	Aqueous humor	ELISA	Higher concentration [58]

*AL* axial length, *ELISA* enzyme-linked immunosorbent assay, *FDM* form-deprivation myopia, *GDF-15* growth differentiation factor 15, *HGF* hepatocyte growth factor, *IFN-γ* interferon gamma, *IL* interleukin, *IP-10* IFN-γ-induced protein 10, *MCP-1* monocyte chemotactic protein 1, *MFD* monocular form deprivation, *MMP-2* matrix metalloproteinase-2, *PDGF-AA* platelet-derived growth factor-AA, *PCR* polymerase chain reaction, *PLG* plasminogen, *sICAM-1* soluble intercellular adhesion molecule 1, *TGF-β* transforming growth factor-beta, *TNF-α* tumor necrosis factor-alpha

Regarding growth factors related to inflammation, TGF-β2 expression in aqueous humor is significantly higher and positively correlated with axial length (AL), suggesting a potential marker for myopia progression [48, 49]. Additionally, growth differentiation factor 15 (GDF-15), hepatocyte growth factor (HGF), and platelet-derived growth factor (PDGF)-AA were also significantly elevated in the aqueous humor in high myopia patients, indicating novel biomarkers for myopia progression [50].

In terms of inflammatory factors, several studies have verified that IL-6 levels are elevated in highly myopic patients and positively correlated with AL [51, 52]. IL-8 is also elevated and positively correlated with AL in some studies [53]. IL-1β is also significantly increased in high myopia, which may be related to the inflammatory response [52]. The expression of monocyte chemotactic protein-1 (MCP-1) in the aqueous humor of patients with high myopic cataract was significantly higher than in age-related cataract patients [54] and positively correlated with AL [49]. In the vitreous humor, the expression of interferon γ (IFN-γ), IL-6, IFN-γ-induced protein 10 (IP-10), eotaxin, and macrophage inflammatory protein 1α (MIP-1α) was significantly elevated, which may serve as myopia-associated markers [55].

Similar results have been validated in animal studies, where the induction of myopia leads to increased TGF-β and matrix metalloproteinase-2 (MMP-2) expression, accompanied by significant increases in IL-6, IL8, TNF-α and MCP-1 [13, 56]. Notably, some discrepancies exist across studies. Zhu et al. [57] found no significant association between AL and inflammatory cytokines (IL-1β, IL-6 and TNF-α) in the aqueous humor of highly myopic cataract patients. Such discrepancies may stem from differences in sample size, methodology, or inclusion criteria, highlighting the need for further experimental validation.

In patients with progressive myopia, soluble intercellular adhesion molecule 1 (sICAM-1) levels in the aqueous humor were also significantly correlated with AL and could be used as a monitoring biomarker [49]. Plasminogen (PLG) expression in the aqueous humor was significantly upregulated, suggesting that this protein may be involved in axial elongation and the pathogenesis of myopia by regulating collagen degradation [58]. The study of potential biomarkers of myopia may provide new possibilities for monitoring myopia progression.

## Systemic and Local Inflammation in Myopia

Although the eye maintains immune privilege, accumulating evidence suggests that both local and systemic inflammations contribute to pathological alterations, including the development of myopia. Inflammatory processes disrupt ocular homeostasis by altering ECM remodeling, scleral biomechanics, and intraocular signaling pathways, ultimately influencing axial elongation. Various ocular and systemic inflammatory diseases have been implicated in myopia progression through distinct mechanisms. Table 2 provides an overview of these conditions and their proposed pathological links to myopia, which will be further explored in the following sections.

**Table 2:** Summary of inflammatory diseases associated with myopia

Disease	Inflammation type	Potential mechanisms	Key factors	Clinical association with myopia
Ocular surface inflammation	Local	Activation of MAPK and NF-κB RGC apoptosis Increased MMP-2 activity	IL-6, IL-8, TNF-α, MMP-2	AC patients have a 2.35-fold higher incidence of myopia [29]
Uveitis	Local	Ciliary exudation-induced lens convexity changes Elevated IOP Scleral weakening	IL-6	Higher risk of myopia in children under 18, especially under 12. JIA-associated uveitis linked to myopia [56]
Chronic scleritis	Local	ECM degradation Scleral weakening	Collagen, proteoglycans	Alters scleral biomechanics, leading to axial elongation. Acute inflammation can induce transient myopia [14, 18]
KD	Systemic	Systemic vasculitis BRB breach MMP-2 activation	IL-1β, TNF-α, MMP-2	KD patients have a higher risk of myopia due to inflammatory factors [70, 71]
SLE	Systemic	Autoimmune dysregulation; chronic systemic inflammation	IL-6, TNF-α	Higher myopia risk in adolescents, especially under 12. Thinner choroidal thickness in JSLE patients [56, 72, 73]
VKH syndrome	Systemic	Ciliary detachment; supraciliary exudation; systemic inflammation	IL-1β, IL-6, TNF-α	Hyperacute VKH results in acute myopia. Myopic progression with increasing AL [74–76]
T2DM	Systemic	High-glucose-induced BRB dysfunction; hypoxia-mediated factors	TGF-β, IL-1β, IL-6, TNF-α, MMP-2	Higher myopia risk, especially in adolescents. Hypoxia exacerbates scleral remodeling [77–82]

*AC* allergic conjunctivitis, *AL* axial length, BRB blood-retinal barrier, *ECM* extracellular matrix, *IL* interleukin, *IOP* intraocular pressure, *JIA* juvenile idiopathic arthritis, *JSLE* juvenile systemic lupus erythematosus, *KD* Kawasaki disease, *MAPK* mitogen-activated protein kinase, *MMP-2* matrix metalloproteinase-2, *NF-κB* nuclear factor kappa B, *RGC* retinal ganglion cell, *SLE* systemic lupus erythematosus, *T2DM* type 2 diabetes mellitus, *TGF-β* transforming growth factor-beta, *TNF-α* tumor necrosis factor-alpha, *VKH* Vogt–Koyanagi–Harada

### Inflammatory Diseases of the Eye Associated with Myopia

Although the eye is immune privileged, accumulating evidence suggests that both local and systemic inflammations contribute to pathological changes, including the development of myopia. Ocular surface inflammation, including dry eye, keratitis, and allergic conjunctivitis (AC), has been shown to activate mitogen-activated protein kinase (MAPK) and NF-κB signaling pathways, which are involved in regulating RGC apoptosis [59]. NF-κB activation can induce the expression of IL-6, IL-8, and TNF-α, which subsequently increase scleral MMP-2 activity, promoting ECM remodeling and axial elongation [15]. A strong association between AC and myopia has been widely reported, with AC patients being 2.35 times more likely to develop myopia than non-AC individuals [29].

Uveitis, one of the common inflammatory diseases of the eye, can cause acute, transient, or constitutive myopia through several mechanisms [14]. In acute anterior uveitis, ciliary exudation-induced relaxation of zonular fibers can lead to lens convexity changes, inducing transient myopia [60]. A cohort study found that children under 18 years old with uveitis had a significantly higher risk of developing myopia, with the risk being even greater in those younger than 12 years [56]. In patients with juvenile idiopathic arthritis (JIA)-associated uveitis, myopia appears more frequently, potentially due to uveitis-induced elevated intraocular pressure (IOP), which weakens the scleral connective tissue, leading to structural instability and axial elongation [61]. Furthermore, IL-6, a key inflammatory mediator, has been shown to be upregulated in both human uveitis patients and experimental myopia models, where it plays a crucial role in ocular growth regulation [51, 62]. Apart from anterior uveitis, multiple posterior uveitic conditions, such as punctate inner choroidopathy [63] and multiple evanescent white dot syndrome[64], are also more commonly observed in myopic eyes.

Chronic scleritis, on the other hand, has a profound impact on myopia progression by altering the biomechanical properties of the sclera. Inflammatory processes within the scleral tissue trigger ECM degradation, including the breakdown of collagen and proteoglycans [18]. This weakens the scleral structure, making it more susceptible to intraocular pressure and mechanical forces, ultimately leading to abnormal axial elongation. Additionally, acute scleral inflammation and choroidal disorders can induce acute myopia through ciliary exudation-mediated relaxation of the zonular fibers, increasing lens convexity [14].

### Myopia and Systemic Inflammatory Diseases

Recent studies have found that patients with high and pathologic myopia exhibit elevated peripheral blood leukocyte counts [65], higher neutrophil-to-lymphocyte ratios [66] and imbalanced platelet-lymphocyte ratios [67], suggesting myopia patients may have a systemic inflammatory status.

Kawasaki disease (KD) is a systemic inflammatory disease primarily involving children under 5 years of age [68]. KD manifests primarily as systemic vasculitis of medium-sized arteries and can involve the eye, presenting as conjunctivitis, iridocyclitis, keratitis, and uveitis [69]. A 2017 cohort study found that KD patients had a significantly increased risk of developing myopia [70]. While the exact mechanism remains unclear, this association may be related to the immuno-inflammatory response observed in KD. Supporting this, a population-based study found that KD patients treated with intravenous immunoglobulin had a lower risk of developing myopia than those treated with aspirin alone, potentially reflecting more effective modulation of immune and inflammatory responses during treatment [71]. However, further studies are needed to clarify the underlying biological mechanisms.

The systemic inflammatory contribution to refractive changes is further supported by the presence of myopia in systemic lupus erythematosus (SLE), an autoimmune inflammatory disease marked by dysregulated immune system activation and chronic systemic inflammation. Acute myopia may represent an early ocular manifestation of SLE [72]. The risk of developing myopia is notably higher in adolescents with SLE (< 18 years old) and even greater in pediatric patients (< 12 years old) [56]. Another clinical study in healthy individuals reported that more severe myopia and longer axial length (AL) were significantly associated with lower macular choroidal thickness values [73]. These findings suggest that systemic autoimmune responses may be involved in myopia pathology through an “inflammation-structural remodeling” pathway, and the stronger association observed in pediatric patients implies a potential age-related vulnerability to inflammation-induced ocular changes.

Vogt-Koyanagi-Harada (VKH) syndrome offers another perspective of inflammation-mediated acute refractive changes. Hyperacute VKH disease was found to frequently result in acute myopia due to ciliary detachment and inflammatory supraciliary exudation [74]. Takahashi et al. also reported that myopic progression occurs with increasing AL in VKH disease. Sunset glow fundus was observed more frequently in patients with VKH, and thinner choroidal thickness was found in the subcentral recess [75]. Notably, IL-1β, IL-6, and TNF-α levels were significantly elevated in the peripheral blood mononuclear cells of patients with VKH [76], which may play a pivotal role in mediating systemic inflammation-induced axial elongation.

Type 2 diabetes mellitus (T2DM) provides insight into the impact of metabolic inflammation on myopia. T2DM is a common systemic inflammatory disease accompanied by elevated inflammatory factors such as TGF-β, IL-1β, IL-6, and TNF-α [77]. T2DM patients are more prone to myopia and related disorders [78], especially adolescent patients under 18, who are prone to myopia and astigmatism due to the fragility of ocular tissues. The high-glucose environment in diabetes promotes peripapillary cell loss and glycocalyx degradation, leading to BRB dysfunction and an increase in inflammatory responses [79]. Clinical studies have also shown that diabetic patients exhibit a significant reduction in the partial pressure of oxygen in the lens and vitreous cavity [80], with corresponding elevations in hypoxia-mediated factors in the preretinal membrane of diabetic animal models [81]. Elevated hypoxia-induced IL-6 has been demonstrated to exacerbate myopia by promoting scleral remodeling through the TGF-β1/Smad2/MMP-2 pathway [82], supporting the hypothesis that metabolic inflammation contributes to myopia through a “metabolic-immune-hypoxia” network. A summary of inflammatory diseases associated with myopia is presented in Table 2.

## The Bidirectional Impact Between Myopia and Inflammation

### Inflammatory Regulation of Myopia Progression

Understanding the inflammatory regulatory mechanisms is essential for elucidating the role of inflammation in myopia progression. Myopia development is closely associated with scleral remodeling, and MMP-2 has been identified as a key enzyme involved in ECM remodeling. Elevated MMP-2 levels reduce type I collagen (COL-1) expression, promoting collagen remodeling and axial elongation [83]. MMP-2 levels are regulated by a variety of inflammation-related factors, including TGF-β, IL-6, and TNF-α. These inflammatory factors are significantly elevated in both animal models and clinical samples of myopia, and they activate inflammatory mediators, such as TNF-α and IL-6, primarily through the NF-κB signaling pathway [84]. This activation, in turn, regulates MMP-2 expression, promotes collagen degradation, and results in scleral thinning. Furthermore, TNF-α may exacerbate myopia progression and NF-κB activation by triggering paracrine feedback loops in the retina or sclera [56].

The NF-κB signaling pathway is not only directly involved in myopia progression, but also acts synergistically with the TGF-β signaling pathway to regulate scleral remodeling [85]. The TGF-β signaling pathway can increase MMP-2 through Smad-dependent and nondependent pathways, and regulates scleral remodeling through collagen metabolism [86–88]. In addition, a polymorphism at the codon 10 locus of the TGFβ1 gene was strongly associated with genetic susceptibility to high myopia [89].

Transcription factors associated with the NF-κB pathway also include activator protein 1 (AP-1). AP-1 is a dimeric transcription factor consisting of Jun and Fos family proteins that can be activated through the mitogen-activated protein kinase (MAPK) pathway, including extracellular signal-regulated kinase (ERK), c-Jun N-terminal kinase (JNK), and p38 MAPK [90]. The MAPK pathway regulates the inflammatory factors by phosphorylating the constituent proteins of AP-1 [91, 92]. The significant overlap in the target genes of NF-κB and AP-1 suggests a cooperative role in inflammation-driven myopia progression [93]. Similarly, MAPK signaling shares activation targets with NF-κB, further supporting their shared role in myopia pathology [94].

In addition to MMP-related mechanisms, protein-associated inflammatory immune modulation is also associated with myopia development. Analyses from patient samples and animal models revealed that the S100 protein-related signaling pathway is significantly upregulated in myopia [95]. Furthermore, upregulation of S100 signaling has been shown to reduce inflammation, promote tissue repair, and help control myopia progression [95]. Similarly, granzyme A in the retina was found to be activated in the lens-induced myopia (LIM) model [96], which may be involved in myopia progression by activating cellular caspase-independent apoptosis [97] and modulating inflammation [98].

The classical and alternative pathways of the complement system have been partially activated in myopia models [99] and are strongly associated with ocular structural damage and scleral remodeling. Activation of the complement system in normal subjects prevents inflammation from causing overstimulation and damage [100]. Elevated levels of C1q, C3, and C5b-9 in the sclera of myopic guinea pig models suggest that complement system activation may induce ECM remodeling [101]. Recent studies have also found that CD55, also known as decay-accelerating factor, can inhibit myopia development by downregulating complement activation and inflammation through the inhibition of complement 3 convertase [102].

Immune cells, particularly macrophages, play a key role in the onset and progression of myopia. Macrophages can enhance local immune responses by secreting pro-inflammatory cytokines, including TGF-β [103], which promote scleral deformation and ocular axial elongation during myopia progression [104]. This has been verified in animal experiments, where increased density and expression of M2-type macrophages in the sclera of a form-deprivation myopia (FDM) mouse model were inhibited using LBH589 to reduce the development of FDM. Gene ontology and Kyoto Encyclopedia of Genes and Genomes pathway analyses revealed that M2 macrophages may regulate ECM remodeling in primary human scleral fibroblasts (HSF) and contribute to myopia progression [105].

**Fig. 3:**
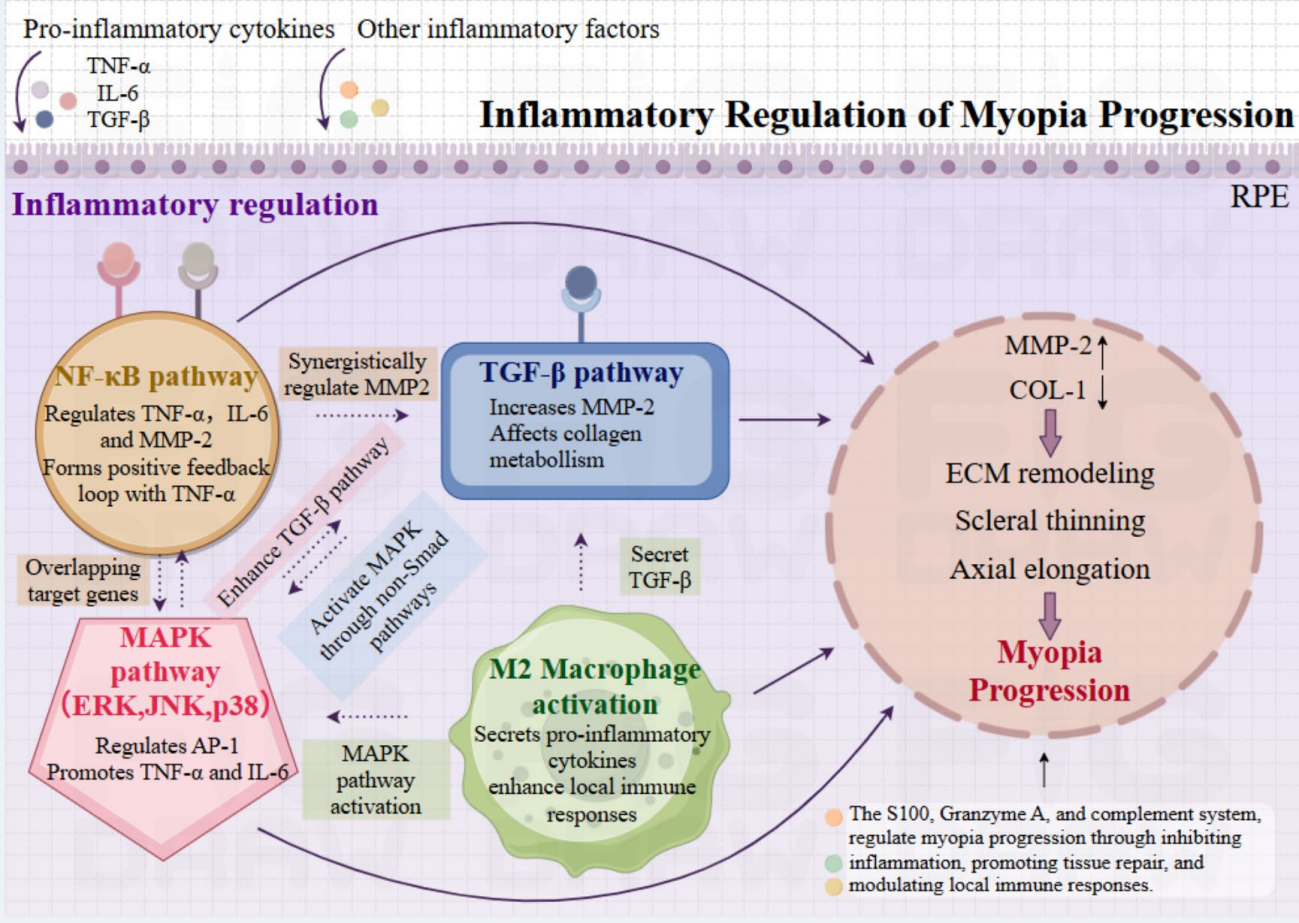
Overview of inflammatory regulation of myopia progression. Key signaling pathways, including NF-κB, MAPK, and TGF-β, are activated by inflammatory cytokines such as TNF-α and IL-6, leading to increased MMP-2 expression and decreased type I collagen (COL-1), which together contribute to ECM remodeling. These pathways interact synergistically to amplify inflammatory signaling and promote scleral thinning and axial elongation. Complement activation and activated M2 macrophages further contribute to myopia progression through local immune activation and TGF-β secretion. Additional immune modulators, such as S100 signaling, the granzyme A pathway, and CD55-mediated complement suppression, are also implicated in inflammation-mediated regulation of myopia. These immune mechanisms interact to form a complex regulatory network that amplifies or mitigates pathological changes during myopia progression. Illustration created with Figdraw. Abbreviations: AP-1, activator protein-1; COL-1, collagen type I; ECM, extracellular matrix; ERK, extracellular signal-regulated kinase; IL-6, interleukin-6; JNK, c-Jun N-terminal kinase; MAPK, mitogen-activated protein kinase; MMP-2, matrix metalloproteinase-2; NF-κB, nuclear factor kappa B; RPE, retinal pigment epithelium; TGF-β, transforming growth factor-beta; TNF-α, tumor necrosis factor-alpha

In summary, immune mechanisms play a crucial role in the development of myopia, with multiple immune pathways contributing to ocular axial elongation and scleral remodeling. Figure 3 provides a schematic overview of inflammatory signaling pathways and immune regulatory networks implicated in myopia progression. As illustrated, activation of cytokine cascades, complement pathways, and immune cell infiltration perpetuates this process, creating a vicious cycle that accelerates myopia progression.

### Interaction Between Inflammation and Myopia

Inflammation plays a critical role in the development of myopia, with research suggesting that ocular hyperextension may further exacerbate the inflammatory response. Lengthening of the ocular axis is a central pathological feature in myopia development, highlighting the close interplay between structural alterations and inflammation. As the ocular axis lengthens, increased traction forces on the posterior eye lead to mechanical damage to tissues such as the retina, choroid, and uvea. This mechanical damage can disrupt ocular tissue homeostasis, activate local inflammatory responses, and trigger a cascade of immune reactions, further exacerbating ocular pathology.

The microvascular systems of the retina and choroid play a crucial role by supplying oxygen and nutrients to both the retina and sclera. Ocular overextension has been shown to lead to thinning of the choroid and microvascular damage, which impairs choroidal blood circulation and reduces the supply of nutrients and oxygen to scleral tissues [106]. A study on human scleral fibroblasts verified that hypoxia-induced IL-6 has been shown to regulate fibroblast proliferation, differentiation, and apoptosis through the TGF-β1/Smad2/MMP-2 pathway, causing scleral remodeling [82]. In addition, inflammatory factors may exacerbate local inflammation by disrupting the tight junctions between corneal epithelial cells and promoting the secretion of TNF-α, IL-6, and IL-8 by RPE cells [29].

Inflammatory modulation of lens refractive power may further accelerate myopic progression. Inflammatory responses in uveitis or the ciliary body can lead to uveal effusion or ciliary body swelling, which affects the suspensory ligaments and ciliary muscles, thus increasing the lens’s refractive power and accelerating myopia onset [15]. Although this refractive change may be reversible in the short term, prolonged inflammatory stimulation may lead to irreversible changes in lens morphology. Both MAPK and NF-κB pathways contribute to ocular surface inflammation and RGC apoptosis [59, 107]. Inflammatory factors drive the production of pro-inflammatory cytokines by triggering the MAPK and NF-κB pathways. Activation of MMP-2 expression in the retina leads to collagen cleavage, scleral remodeling, and finally myopia. Myopia, in turn, enhances MMP-2 production, further amplifying the release of inflammatory factors and perpetuating the vicious cycle.

**Fig. 4:**
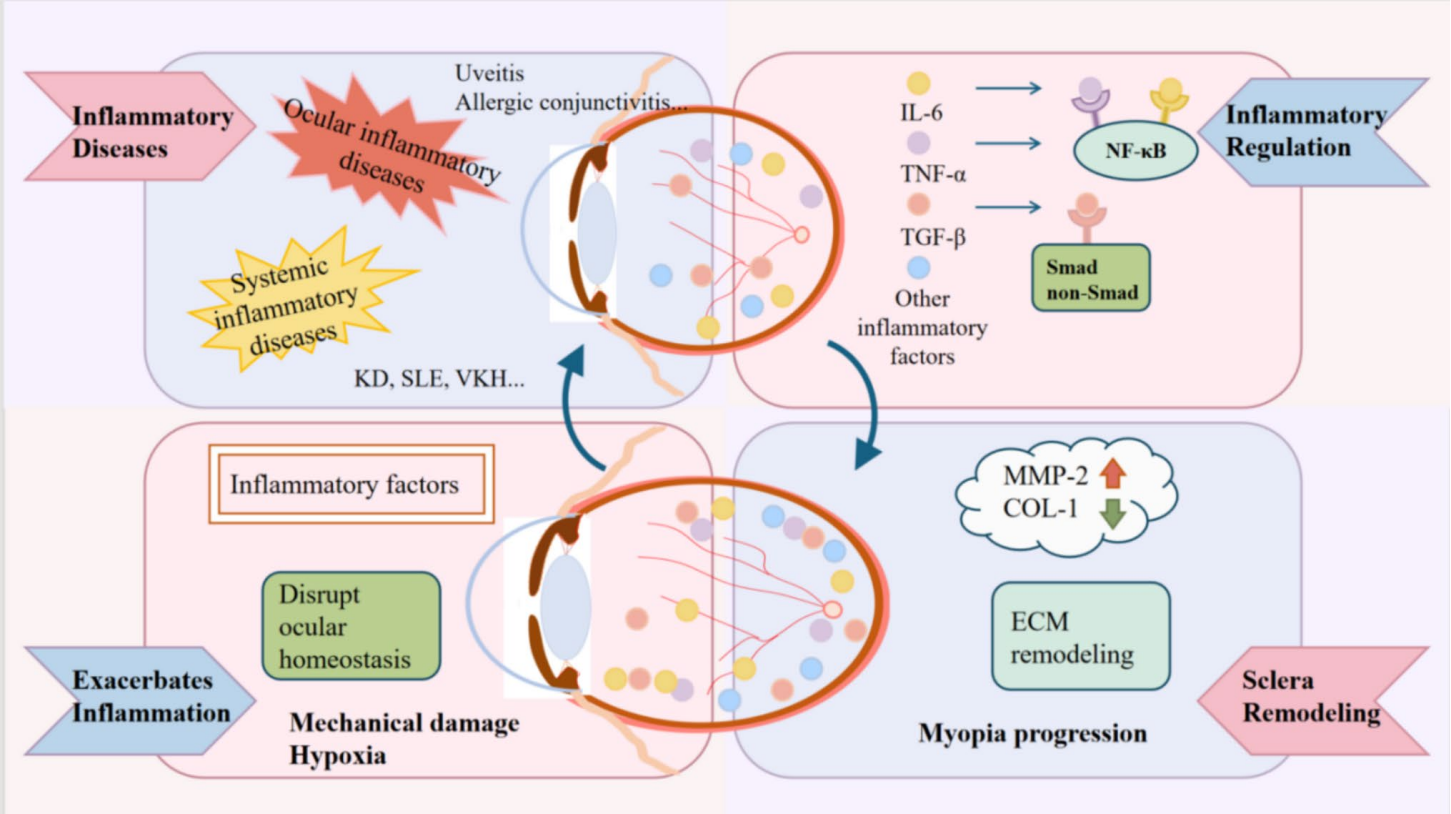
Bidirectional interaction between inflammation and myopia progression. Overview of the reciprocal relationship between inflammation and myopia progression. Inflammatory factors, including TNF-α, IL-6, and TGF-β, activate signaling pathways such as NF-κB, promoting extracellular matrix (ECM) remodeling, scleral thinning, and axial elongation. In parallel, ocular elongation and associated mechanical stress exacerbate local inflammation by inducing hypoxia, microvascular damage, and immune cell activation. This feedback loop perpetuates the progression of myopia, underscoring the pivotal role of inflammation in its pathogenesis. Abbreviations: COL-1, type I collagen; ECM, extracellular matrix; IL-6, interleukin-6; KD, Kawasaki disease; MMP-2, matrix metalloproteinase-2; NF-κB, nuclear factor kappa B; SLE, systemic lupus erythematosus; Smad, SMAD signaling proteins; TGF-β, transforming growth factor-beta; TNF-α, tumor necrosis factor-alpha; VKH, Vogt-Koyanagi-Harada syndrome

The inflammatory state in myopic eyes is more pronounced, suggesting that chronic inflammation may play an important role in the onset and progression of myopia. The levels of inflammatory factors in the aqueous humor and vitreous are significantly elevated in high myopia patients, and these changes are strongly correlated with the increase in the AL [51, 55]. The accumulation of inflammatory factors not only exacerbates uveitis, retinitis, and scleritis but also may alter the biomechanical properties of the eye and further elongate the AL. Based on these findings, it is evident that inflammation and myopia progression are interconnected through a self-perpetuating cycle. To illustrate this bidirectional interaction, the key inflammatory mechanisms and downstream ocular changes are summarized in Fig. 4.

In addition, oxidative stress has emerged as a pivotal mediator linking inflammation and myopia progression. Reactive oxygen species (ROS) generated in ocular tissues can activate proinflammatory signaling cascades, such as the NF-κB pathway, leading to the upregulation of cytokines like TNF-α and IL-6, which contribute to scleral remodeling and axial elongation [108]. Studies using FDM models have demonstrated increased oxidative stress markers and reduced antioxidant enzyme activity in the retina and RPE, suggesting that oxidative stress exacerbates retinal inflammation and structural disruption [109, 110]. Moreover, ROS can directly impair extracellular matrix homeostasis in the sclera, promoting tissue thinning and elongation of the globe [111]. Given the bidirectional relationship between oxidative stress and inflammation, targeting oxidative pathways may offer novel therapeutic strategies to slow or reverse myopia progression.

Thus, in patients with high myopia, the interaction between inflammation and myopia creates a self-perpetuating cycle. Inflammatory factors promote scleral remodeling and accelerate ocular axis elongation, which in turn exacerbates mechanical stress and activates inflammatory signaling pathways that perpetuate inflammation. This feedback loop may represent a self-reinforcing mechanism; however, the current evidence primarily reflects associative trends, and further research is necessary to determine whether a direct causal relationship exists.

## Advances in Myopia-Related Anti-inflammatory Therapy

Given that the progression of myopia is closely linked to inflammation, recent research has focused on the possibility of controlling AL through anti-inflammatory treatments. Various anti-inflammatory drugs have shown potential in halting myopia progression by downregulating pro-inflammatory mediators, inhibiting collagen degradation, and modulating the ocular immune microenvironment. Recent advances in anti-inflammatory therapies are summarized in Table 3.

**Table 3:** Potential anti-inflammatory treatments for myopia

Treatments	Sample source	Mechanism	Therapeutic effects
Atropine	MFD model	Reduces expression of inflammatory factors (c-Fos, NF-κB, IL-6, TNF-α) Promotes COL-1 expression	Reduces inflammation Inhibits axial elongation [56, 113]
CSA	MFD model	Reduces expression of c-Fos, IL-6, TNF-α, and NF-κB Increases IL-10 immunoreactivity	Reduces inflammation Mitigates refractive changes [56]
Diacerein	MFD model	Blocks AKT and NF-κB pathways Inhibits TGF-β1 and MMP-2 activation Reduces IL-6, IL-8, and MCP-1 Promotes COL-1 expression	Slows myopia progression Inhibits axial elongation [119, 120]
Resveratrol	MFD model	Inhibits TGF-βand NF-κB Reduces TNF-α, IL-1β, and IL-6 Promotes COL-1 expression	Reduces inflammation Slows myopia progression [122, 123]
FJE + PVE	MFD model	Suppresses Akt and NFκB-mediated inflammatory reactions Promotes COL-1 expression	Reduces inflammation Inhibits axial elongation [126]
Lactoferrin	LIM model	Inhibits MMP-2 expression Activates ERK1/2 signaling pathway	Reduces inflammation Digested LF and holo-LF inhibit axial elongation [130]
RLRL	DTH model	Reduces TNF-α, IFN-γ, and IL-10	Reduces retinal inflammation [133]
RLRL	Patient cohort	Improves choroidal blood perfusion and circulation	Controls AL and spherical equivalent Slows myopia progression [134]

*Akt* protein kinase B, *AL* axial length, *COL-1* type I collagen, *CSA* Cyclosporine A, *DTH* delayed-type hypersensitivity, *ECM* extracellular matrix, *ERK1/2* extracellular signal-regulated kinase 1/2, *FJE Fallopia japonica* extract, *IL* interleukin, *LIM* lens-induced myopia, *LF* lactoferrin, *MCP-1* monocyte chemoattractant protein-1, *MFD* monocular form deprivation, *MMP-2* matrix metalloproteinase-2, *NF-κB* nuclear factor kappa B, *PVE Prunella vulgaris* extract, *RLRL* repeated low-level red-light, *TGF-β* transforming growth factor-beta, *TNF-α* tumor necrosis factor-alpha

Atropine is a well-established pharmacological agent for myopia control, with mechanisms involving muscarinic receptor antagonism and dopaminergic signaling [112]. In addition to its neuromodulatory effects, a 2025 study showed that atropine improves choroidal perfusion and reduces hypoxia-related remodeling in the choroid and sclera [113]. Furthermore, recent experimental studies have reported that atropine reduces inflammatory markers such as c-Fos, NF-κB, IL-6, and TNF-α in form-deprivation myopia models, while promoting COL-1 expression [56]. Notably, independent studies in systemic inflammatory models have also demonstrated that atropine reduces TNF-α, elevates IL-10, and improves survival following LPS-induced endotoxemia, suggesting its broader anti-inflammatory potential beyond ocular contexts [114]. However, whether its clinical efficacy in myopia control is mediated through anti-inflammatory mechanisms remains to be fully elucidated.

Cyclosporine A (CSA) is a calcineurin inhibitor that suppresses T cell activation and cytokine transcription via NFAT signaling [115]. It is a clinically established anti-inflammatory agent in DED, with comparable efficacy demonstrated for 0.05% and 0.1% concentrations in postoperative inflammation after cataract surgery [116]. Beyond T cell regulation, CSA also modulates innate immune responses and has shown anti-inflammatory efficacy in models of autoimmune uveitis [117] and acute inflammation [118]. In FDM models, CSA treatment mitigated refractive changes; downregulated c-Fos, IL-6, TNF-α, and NF-κB; and enhanced IL-10 expression [56], supporting its potential in inflammation-targeted myopia therapy, although further clinical validation is needed.

Diacerein, an anthraquinone derivative, inhibits inflammation by targeting the IL-1β pathway and blocking downstream MEK/ERK and NF-κB signaling [119]. In a hamster monocular form deprivation (MFD) model, 10 mM diacerein effectively inhibited TGF-β1 and MMP-2 activation, decreased IL-6, IL-8, and MCP-1 expression, and increased COL-1 expression, thereby slowing AL [120]. Additionally, in patients with DED, oral diacerein significantly improved corneal staining scores and markedly reduced tear IL-1β levels, indicating ocular surface anti-inflammatory activity [121]. These findings highlight the therapeutic potential of diacerein in inflammation-targeted myopia interventions; however, further clinical validation is warranted.

Antioxidant drugs have also garnered attention for their anti-inflammatory properties, with resveratrol being one of the most studied. As a natural antioxidant, resveratrol reduces inflammatory factors like TNF-α, IL-1β, and IL-6 by inhibiting the NF-κB and MAPK pathways [122]. In the MFD model, resveratrol enhanced COL-1 expression and inhibited MMP-2 and TGF-β levels [123]. Kubota et al. also reported oral resveratrol reduced ICAM-1 and MCP-1 expression in an endotoxin-induced uveitis model [124], supporting its anti-inflammatory effects in ocular tissues. Moreover, resveratrol has been evaluated in ocular diseases such as age-related macular degeneration and diabetic retinopathy, where it showed potential in reducing vascular endothelial growth factor (VEGF) levels and improving retinal function [125]. Despite these results, no clinical trials have assessed its efficacy in myopia, and its low bioavailability remains a barrier to translation.

The combined use of the phytochemicals FJE (*Fallopia japonica* extract) and PVE (*Prunella vulgaris* extract) has been shown to reduce inflammatory factors such as NF-κB, TGF-β, IL-1β, IL-6, IL-8, and TNF-α, increase COL-1 expression, and inhibit axial elongation [126]. Additionally, artificial exosomes have been demonstrated to decrease pro-inflammatory factor expression [127] and inhibit inflammatory cell infiltration [128], suggesting a potential anti-inflammatory strategy that warrants further investigation.

Lactoferrin (LF), an iron-binding glycoprotein with anti-inflammatory effects, is widely distributed in breast milk, tears, saliva, blood, and neutrophils. It plays an important role in regulating immune responses, inhibiting the release of inflammatory mediators, and enhancing host defenses [129]. A LIM study showed that LF and its hydrolysates significantly reduced IL-8, MMP-2, and NF-κB levels, suggesting that LF may delay myopia progression by attenuating inflammation [130]. Mice treated with digested LF or holo-LF had shorter AL and suppressed myopia more effectively than those given native LF, indicating their potential relevance to myopia treatment, pending further clinical investigation.

In addition to conventional medications, repeated low-level red-light (RLRL) treatment for myopia has gained worldwide attention in recent years. Red-light therapy at 670 nm reduces retinal inflammation by increasing mitochondrial membrane potential [131] and promoting cytochrome C oxidase expression [132]. Studies have also reported that RLRL reduces the release of inflammatory factors and inhibits inflammation [133], which may control the progression of myopia. One-year follow-up data demonstrated that RLRL therapy significantly improved choroidal blood perfusion and circulation, controlled axial elongation, and showed promise as an effective myopia control therapy [134]. Regarding the safety of RLRL therapy in children, it can effectively improve myopia without significant adverse effects on retinal function and structure over 12 months. However, the changes in the relative reflectance of the ellipsoid zone and photoreceptor outer segments still require further investigation to ensure long-term retinal safety [135]. In China, red-light therapy devices for myopia control have been proposed to be regulated as Class II medical devices under the draft guidelines issued by the National Medical Products Administration, which reflects their intended status as approved medical devices.

Overall, anti-inflammatory therapies offer promising new strategies for myopia control, particularly through modulating immune responses and attenuating ocular inflammation. While existing studies provide a strong theoretical basis for anti-inflammatory approaches, clinical translation is still in its infancy and warrants further validation. Future studies should prioritize long-term efficacy and safety assessments, particularly in pediatric and adolescent populations. Moreover, combining genomics, cell biology, and other advanced technologies to uncover the specific mechanisms of inflammation in myopia development will be essential to elucidate the specific immunopathological mechanisms underlying myopia, ultimately paving the way for precision medicine and individualized treatment strategies.

## Conclusions and Future Directions

Emerging evidence highlights a bidirectional relationship between myopia and inflammation, where systemic and ocular inflammatory processes contribute to myopia progression, while excessive axial elongation exacerbates local inflammation. Inflammatory cytokines such as IL-6 and TNF-α disrupt the blood-retinal barrier, activate NF-κB/MAPK pathways, and drive scleral ECM degradation, promoting axial elongation. Meanwhile, progressive axial elongation induces mechanical stress, retinal stretching, and choroidal hypoxia, which activate microglia, dysregulate the complement system, and cause immune imbalances, perpetuating chronic low-grade inflammation and further scleral remodeling. This inflammation-induced structural remodeling, combined with myopia-driven inflammation, may form a reinforcing pathological cycle, although further research is needed to confirm a direct causal link.

Although anti-inflammatory agents like diacerein and resveratrol have shown efficacy in experimental models, challenges such as drug delivery, long-term safety, and variability in inflammatory biomarker responses hinder their clinical use. Additionally, emerging therapies like FJE + PVE combinations and RLRL treatment show potential in modulating ocular inflammation and oxidative stress. Future research should focus on multi-omics approaches to better understand the inflammatory landscape in myopic eyes, optimize targeted drug delivery systems for localized immune modulation, and conduct biomarker-driven clinical trials to personalize treatment strategies. Addressing the inflammation-myopia cycle requires a shift from symptomatic management to mechanistic intervention, with immune regulation offering a promising approach to mitigate myopia progression.

## Data Availability

No datasets were generated or analysed during the current study.

## Abbreviations

AL: Axial Length
COL-1: Collagen Type I
CSA: Cyclosporine A
FJE: Fallopia Japonica Extract
IL-6: Interleukin-6
MMP-2: Matrix Metalloproteinase-2
NF-κB: Nuclear Factor Kappa B
PVE: Prunella Vulgaris Extract
RGC: Retinal Ganglion Cells
RLRL: Repeated Low-level Red Light
RPE: Retinal Pigment Epithelium
SLE: Systemic Lupus Erythematosus
T2DM: Type 2 Diabetes Mellitus
TGF-β: Transforming Growth Factor-beta
TNF-α: Tumor Necrosis Factor-alpha
VKH: Vogt-Koyanagi-Harada syndrome
